# A Simple but Universal Fully Linearized ADMM Algorithm for Optimization Based Image Reconstruction

**DOI:** 10.21203/rs.3.rs-2857384/v1

**Published:** 2023-04-28

**Authors:** Zhiwei Qiao, Gage Redler, Boris Epel, Howard Halpern

**Affiliations:** Shanxi University; Moffitt Cancer Center; University of Chicago; University of Chicago

**Keywords:** fully linearized ADMM, optimization, total variation, computed tomography, image reconstruction

## Abstract

**Background and Objective:**

Optimization based image reconstruction algorithm is an advanced algorithm in medical imaging. However, the corresponding solving algorithm is challenging because the optimization model is usually large-scale and non-smooth. This work aims to devise a simple but universal solver for optimization models.

**Methods:**

The alternating direction method of multipliers (ADMM) algorithm is a simple and effective solver of the optimization models. However, there always exists a sub-problem that has not closed-form solution. One may use gradient descent algorithm to solve this sub-problem, but the step-size selection via line search is time-consuming. Or, one may use fast Fourier transform (FFT) to get a closed-form solution if the system matrix and the sparse transform matrix are both of special structure. In this work, we propose a simple but universal fully linearized ADMM (FL-ADMM) algorithm that avoids line search to determine step-size and applies to system matrix and sparse transform of any structures.

**Results:**

We derive the FL-ADMM algorithm instances for three total variation (TV) models in 2D computed tomography (CT). Further, we validate and evaluate one FL-ADMM algorithm and explore how the two important factors impact convergence rate. Also, we compare this algorithm with the Chambolle-Pock algorithm via real CT phantom reconstructions. These studies show that the FL-ADMM algorithm may accurately solve optimization models in image reconstruction.

**Conclusion:**

The FL-ADMM algorithm is a simple, effective, convergent and universal solver of optimization models in image reconstruction. Compared to the existing ADMM algorithms, the new algorithm does not need time-consuming step-size line-search or special demand to system matrix and sparse transform. It is a rapid prototyping tool for optimization based image reconstruction.

## 1. Introduction

Image reconstruction is the core technique of medical imaging. There are three types of reconstruction algorithms: analytical algorithm [1], optimization based, iterative algorithm [2, 3] and deep learning/machine learning based algorithm [4]. Among them, the optimization based algorithm has some advantages relative to the two other algorithms. Compared to the analytical algorithm, the optimization based algorithm may incorporating prior information into the optimization model and thus may achieve more accurate reconstruction from incomplete data and/or noisy data. Compared to the deep learning based algorithm, the optimization based algorithm is more stable and is not data dependent [5]. Therefore, the optimization based algorithm is always a hot spot in image reconstruction field.

The imaging system model of the optimization based algorithm is a linear system of equations. Usually, it is large-scale, ill-posed and often underdetermined. So, it is impossible to solve the linear system by direct inversion. We may continue to construct an optimization model to solve the ill-posed and underdetermined problems by incorporating prior information like sparse prior [6] or low-rank prior [7], etc. In the optimization models for image reconstruction, total variation (TV) type models have been widely used for they may not only suppress streak artifacts but also suppress noise [8]. In 2006, Sidky *et al.* proposed a data divergence constrained, TV (DDcTV) minimization model for 2D CT [9]. In 2008, Sidky *et al.* proposed a DDcTV model for 3D CT and achieved accurate reconstruction [2]. From then on, a variety of TV-type models were proposed in image reconstruction, for example adaptively weighted TV (awTV) [10], anisotropic TV (aTV) [11], high order TV (HOTV) [12, 13], non-local TV (NLTV) [14], nuclear TV (nTV) [15], directional TV (dTV) [16], etc. These TV-type models may achieve more accurate reconstructions in their suitable cases. However, the solving problems of these TV-type models are challenging because these models are large-scale and non-smooth. They cannot be solved by simple gradient decent algorithm.

For TV-type models, there are mainly three solving algorithms: adaptive steepest descent-projection onto convex sets (ASD-POCS) algorithm [2, 9], Chambolle-Pock (CP) algorithm [17, 18], and alternating direction method of multipliers (ADMM) algorithm [19–26]. We have systematically studied ASD-POCS and CP algorithms and applied them to solve TV models for CT and electron paramagnetic resonance imaging (EPRI) [27–30]. ASD-POCS algorithm is devised according to the physical meaning and the optimization strategy, whereas it is not derived mathematically. Thus, those algorithm parameters in this algorithm should be carefully, empirically chosen to achieve convergence. CP algorithm is obtained by mathematical derivation and may guarantee convergence, but the derivation is not so easy for those who are not familiar with optimization, for example it needs the calculation of convex conjugate [18]. ADMM is another algorithm framework for solving TV-types models. The derivation of algorithm instance according to the ADMM algorithm framework is easier than CP algorithm for the core step is only to split the whole optimization problem into several simple sub-problems by alternating direction technique. However, for TV-types models in image reconstruction, there is always a sub-problem that has not closed-form solution in ADMM algorithm. Thus, this sub-problem is not easy to solve and people began to search techniques to process this issue.

Let’s focus on TV minimization in image denoising, restoration and reconstructions and see how the ADMM-like or ADMM algorithm is used to solve the TV models and how the difficult sub-problem is solved. These models have similar formulation to (20) of this paper. To better describe this algorithm development line, we use the symbols in (20) uniformly.

Now, let us regard (20) as a general image processing model. If *A* is an identity matrix, then it is a model for image denoising.

If its function is to blur an image, then it is an image restoration model. Here, *A* has a special structure for its operation is equivalent to convolution to an image via a blurring mask. In image reconstruction, however, *A* is just a normal matrix without special structure. In (21), *D* is the gradient transform matrix, which has special structure for its operation on an image is equivalent to convolution to an image via a finite-difference mask. For the aim of this work is to devise a simple but universal ADMM-type algorithm, we should note the simplicity and universality of each algorithm below.

In 2007 and 2008, Wang *et al* proposed an alternating minimization algorithm for TV model in image restoration/deblurring [20, 31]. In fact, this algorithm is an ADMM-like algorithm. One may regard it as a simplified ADMM algorithm. This algorithm introduces an auxiliary variable to replace *Du*, uses the penalty technique to ensure the equivalence of the substitution, and then use the variable-splitting technique to solve the TV model. After splitting, there are two sub-problems: one is data-fidelity sub-problem and the other one is TV-regularization sub-problem, which may be solved by the commonly used shrinkage operation. However, the data-fidelity sub-problem has not explicit closed-form solution. Fortunately, in this sub-problem, *A* and *D* are both of special structure for their operations on an image are equivalent to convolution to an image. Thus, this sub-problem may be solved by 2D fast Fourier transforms (FFT). However, we must note that this algorithm cannot be used in image reconstruction for the system matrix, i.e. *A*, has not this special structure.

In 2008, Huang *et al* proposed an alternating minimization algorithm for TV model in image restoration [21]. They introduce a new variable that is equal to the unknown image, then use the penalty technique to formulate the original objective function into the new one. Next, they use alternating minimization to split the problem into two sub-problems. The first one is the data-fidelity sub-problem which may be solved by the FFTs. The second one is the TV-regularization sub-problem which may be solved by the Chambolle’s projection algorithm. However, we must also note that this solving algorithm still cannot be used in image reconstruction for the system matrix has not special structure, i.e, its operation is not equivalent to a convolution operation.

In 2009, Goldstein *et al* proposed the split Bregman algorithm for solving TV model in magnetic resonance imaging (MRI) [22]. Similar to the above solving algorithm, the split Bregman algorithm includes two important sub-problems. One may be solved by FFTs, whereas the other one is solved by shrinkage operation. The advantage of split Bregman algorithm over alternating minimization algorithm is that it has not the penalty parameter whose ideal value is infinite. But, this algorithm still cannot be used in CT and EPRI reconstruction for it utilizes the special structure of the system matrix in MRI reconstruction and use FFTs to solve the data-fidelity sub-problem. Note that the split Bregman algorithm is equivalent to the ADMM algorithm.

In 2010, Yang *et al* proposed the ADMM algorithm for TV models in MRI reconstruction [25]. It is very similar to the Split Bregman algorithm. Each iteration only involves simple shrinkages and FFTs. Still note that it cannot be used in image reconstruction whose system matrix has not special structure.

Clearly, these ADMM-like or ADMM algorithms all have demand to the system matrix so as to use FFTs, so they are not universal solver. To approach a universal solver, people need to explore new techniques.

In 2010, 2011 and 2013, Li *et al* proposed their universal ADMM algorithm for TV model in image reconstruction [26, 32, 33]. They use the nonmonotone line search to decide the step-size of the gradient descent algorithm for solving the data-fidelity sub-problem. Since this is not a closed-form solution, this inner-iteration should be done many times, for which the time-consuming line search is necessary.

In 2012, Xiao *et al* proposed the linearized ADMM (L-ADMM) algorithm for TV model in compressed sensing problems [24]. They linearize the data-fidelity term for solving the corresponding sub-problem and then the FFTs may be used to get a closed-form solution. This algorithm allow the system matrix has a general structure, so it is an universal solver for any type of system matrix. But this algorithm still utilize the special structure of *D*. If *D* is another sparse transform that is not equivalent to convolution operation, then the FFTs cannot be used to achieve closed-form solution.

Clearly, linearized ADMM algorithm has potential to approach universal solver for optimization models in image reconstruction.

In 2012, Chan *et al* proposed an L-ADMM algorithm for constrained linear least-squares problem in image deblurring [34]. They linearized the quadratic regularization term and got a simple closed-form solution for this regularization sub-problem. However, the data-fidelity sub-problem still uses the special structure of the system matrix whose function is blurring so that the closed-form solution may be achieved by FFTs. This algorithm is not a universal solver for image reconstruction. But we may find the potential of L-ADMM to achieve closed-form solution.

In 2013, Yang *et al* proposed an L-ADMM algorithm for nuclear norm minimization [35]. They linearized the data-fidelity term so as to get a closed-form solution.

In 2015, Fang *et al* proposed a linearized generalized ADMM (L-G-ADMM) algorithm and demonstrated its high efficiency [36]. In 2015, Ouyang *et al* proposed an accelerated L-ADMM algorithm whose convergence rate is faster than the L-ADMM [37]. In 2016, Nien *et al* proposed a relaxed L-ADMM algorithm for CT image reconstruction via over-relaxation technique and achieved high-speed iterative reconstruction. The three algorithms may all accelerate the original L-ADMM algorithm [38].

In 2019, Liu *et al* applied the L-ADMM to a non-convex non-smooth optimization problem and achieved good performance [39].

Clearly, the L-ADMM algorithm may simplify the solving problem of the difficult sub-problems in ADMM algorithm for it may construct closed-form solution. However, we found that these L-ADMM algorithms are not thorough, i.e. people only linearized one quadratic term in the difficult sub-problem. Why do not people linearize all the quadratic terms in the difficult sub-problem? We think it should be deeply investigated. By fully linearization, we expect that a simple but universal solver of closed form may be constructed for the difficult sub-problem in ADMM algorithm instance. ‘universal’ means that the algorithm does not demand the special structure of the system matrix and the sparse-transform matrix. ‘simple’ means that the core operations only involve simple matrix-vector multiplications and some simple closed-form operations like shrinkage or projection.

In this work, we proposed a fully linearized ADMM (FL-ADMM) for image reconstruction to simplify the ADMM algorithm by avoiding the search of optimal step-size in gradient descent algorithm and the use of FFT algorithm. The proposed FL-ADMM algorithm framework may be used to derive simple, effective, universal, and convergent algorithm instances for a variety of optimization models in image reconstruction.

To show the potential of the FL-ADMM for prototyping of the optimization models, we derive the FL-ADMM algorithm instances for unconstrained TV (uTV) minimization model, data divergence constrained, TV (DDcTV) minimization model, and TV constrained, data divergence minimization (TVcDM) model for two dimensional (2D) computed tomography (CT).

Also, we validate and evaluate the DDcTV-FL-ADMM algorithm for 2D CT to illuminate that the FL-ADMM algorithm is actually an accurate solver of the DDcTV model. In addition, we explore how the penalty parameter and other algorithm parameters of DDcTV-FL-ADMM impact the convergence rate. Finally, we compare the algorithm with another established universal solver, CP algorithm, to demonstrate its performance.

In Section 2, we give the derivation of the FL-ADMM algorithm instances for three TV models. In Section 3, we perform reconstruction experiments via the proposed DDcTV-FL-ADMM algorithm. We give deep discussions and draw brief conclusions in Sections 4 and 5, respectively.

## 2. Methods

### 2.1 Preliminary knowledge

In this section, we give some basic optimization knowledge, which will be used in the following parts.

#### 2.1.1 Shrinkage algorithm

##### (1) One dimensional (1D) shrinkage

1D Shrinkage algorithm or operation may solve the optimization problem shown in Eq. (1).


(1) $$x^{*}=\arg\underset{x}{\min}\lambda {\left\|x\right\|}_{1}+\frac{1}{2}{\left\|x-a\right\|}_{2}^{2}$$

Here, *x* is a vector of size *N*, ${\left\|•\right\|}_{1}$ is the *l*_1_ norm a vector, and ${\left\|•\right\|}_{2}$ is the *l*_2_ norm of a vector.

Suppose that *x*_*i*_ indicates the *i*th element of this vector. Then the solution of this optimization problem is

(2) $${x_{i}}^{*}=\text{S}\left(a_{i},\lambda \right)=\max\left(\left|a_{i}\right|-\lambda ,0\right)•\mathrm{sgn}\left(a_{i}\right)1\leq i\leq N$$

Here, S is the 1D shrinkage operator and $\mathrm{sgn}\left(•\right)$ is the standard sign function, whose value is 1 for positive number, 0 for 0, and − 1 for negative number. $\max\left(•,•\right)$ is an operator for selecting maximal value.

##### (2) Two dimensional (2D) shrinkage

2D Shrinkage algorithm or operation may solve the optimization problem shown in Eq. (3).


(3) $$x^{*}=\arg\underset{x}{\min}\lambda {\left\|{\left\|x\right\|}_{2}\right\|}_{1}+\frac{1}{2}{\left\|x-a\right\|}_{2}^{2}$$

Here, *x* is a 2D-vector-valued vector of size *N*, *x*_*i*_ indicates the *i*th element of this vector, $x_{i}^{1}$ and $x_{i}^{2}$ are the two elements of the 2D vector, *x*_*i*_.

The solution of this optimization problem is

(4) $${x_{i}}^{*}={\text{S}}^{2}\left(a_{i},\lambda \right)=\max\left({\left\|a_{i}\right\|}_{2}-\lambda ,0\right)•a_{i}/{\left\|a_{i}\right\|}_{2}1\leq i\leq N$$

Here, S^2^ is the 2D shrinkage operator and we should note that *x*_*i*_ and *a*_*i*_ are both 2D vector.

#### 2.1.2. Projection algorithm

In optimization theory, projection onto convex sets (POCS) algorithm/operation may solve a special optimization problem as follows.


(5) $$x^{*}=\arg\underset{x}{\min}{\left\|x-a\right\|}_{2}^{2}s.t.x\in C$$

Here, *C* is a convex set.

Then, the solution of this optimization problem is

(6) $$x^{*}=\text{P}\left(a,C\right)$$

Here, P is the POCS operator. The meaning of (6) is to project the point *a* onto the convex set *C*.

##### 1. Projection onto *l*_2_ norm ball

If the convex set is a *l*_2_ norm ball of radius, *r*, then the POCS operation of *a* to this ball is

(7) $$\text{P}\left(a,L2Ball\left(r\right)\right)=\frac{ra}{\max\left(r,{\left\|a\right\|}_{2}\right)}.$$

Here, *L*2*Ball* (*r*) is the *l*_2_ norm ball of radius *r*. This POCS operation means that, if the point *a* is in the ball, then the projection value is *a*, whereas, if the point is outside the ball, then projection value is the intersection point of this *l*_2_ norm circle and the vector *a* (Note that any point may also be regarded as a vector.)

##### (2) Projection onto *l*_1_ norm ball

**Fig. 2:**
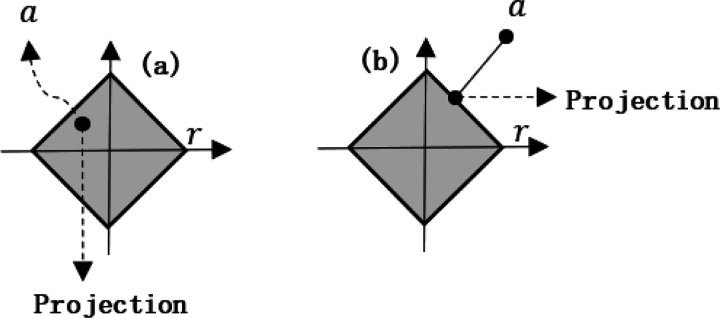
Projection onto *ℓ*_1_ norm ball. (a) is for the case that *a* is in the ball, and (b) is for the case that *a* is outside the ball.

From Fig. 2, we may see that, if *a* is in the *l*_1_ norm ball, the projection point is still itself, whereas, if it is outside the ball, the projection point should be the foot of a perpendicular from *a* to the *l*_1_ norm circle.

There exists accurate algorithm for P (*a*, *L*1*Ball* (*r*)), shown in Algorithm 1 [40].

Algorithm 1

Pseudo-codes for


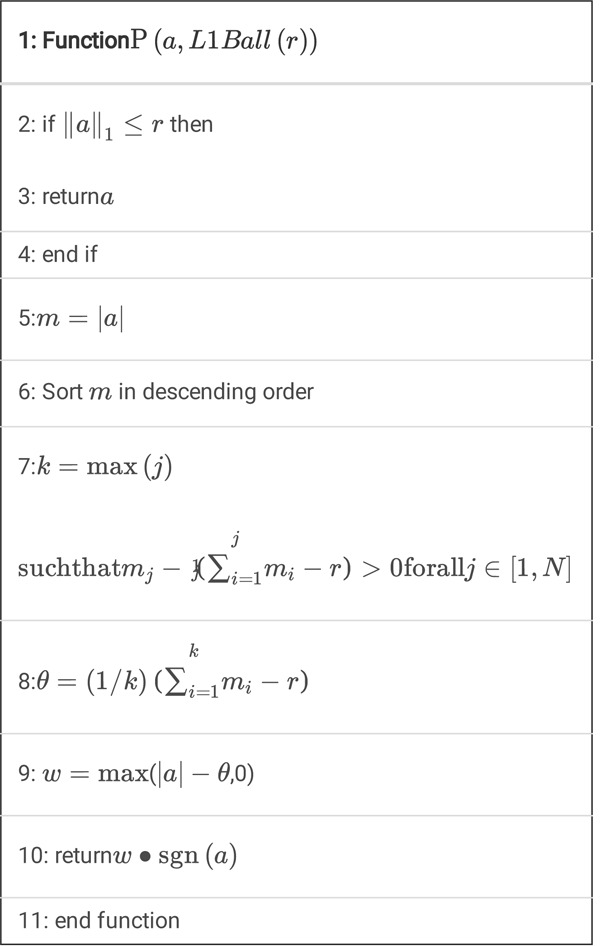


### 2.1.3. Indicator function and constrained-unconstrained transformation

The indicator function may be defined as

(8) $$\delta_{C}\left(x\right)=\left\{\begin{array}{c} 0x\in C\\ \infty x\notin C \end{array}\right.$$

Thus, a constrained optimization model may be transformed into the unconstrained optimization form. For example, Eq. (5) may be written in the unconstrained form as follows.


(9) $$x^{*}=\arg\underset{x}{\min}{\left\|x-a\right\|}_{2}^{2}+\delta_{C}\left(x\right)$$

In the following sections, we will often use this transformation. Note that, it is just a form-transformation and the optimization meaning of the two forms are completely the same.

### 2.1.4. Linearization of a quadratic function

In fact, any quadratic function may be linearized at a point according to the Taylor expansion.


(10) $$\frac{\beta }{2}{\left\|Ax-b\right\|}_{2}^{2}\approx \frac{\beta }{2}{\left\|Ax_{0}-b\right\|}_{2}^{2}+<x-x_{0},\beta A^{T}\left(Ax_{0}-b\right)>+\frac{\text{s}}{2}{\left\|x-x_{0}\right\|}_{2}^{2}$$

Here, *x*_0_ is any point (vector), s cannot be selected arbitrarily and usually we may set its value according to [36]

(11) $$\text{s}\geq \beta {\left\|A^{T}A\right\|}_{2}=\beta \lambda_{max}\left(A^{T}A\right).$$

Here, *λ*_*max*_ means the largest eigenvalue of a matrix.

## 2.2. Three ADMM algorithm framework

### 2.2.1 ADMM algorithm framework

We consider the structured constrained convex optimization problem

(12) $$\min\left\{\theta_{1}\left(x\right)+\theta_{2}\left(y\right)\right\}s.t.\;Ax+By=b$$

Where, *x* and *y* are multi-dimensional vectors, *A* and *B* are matrices indicating linear transform, *θ*_1_ (*x*) and *θ*_2_ (*y*) are convex but not necessary smooth functions.

The corresponding augmented Lagrange function is

(13) $$L_{\beta }\left(x,y;\lambda \right)=\theta_{1}\left(x\right)+\theta_{2}\left(y\right)-<\lambda ,Ax+By-b>+\frac{\beta }{2}{\left\|Ax+By-b\right\|}_{2}^{2}$$

The ADMM algorithm framework is

(14.1) $$\left\{\begin{array}{c} x^{k+1}=\arg\min L_{\beta }\left(x,y^{k};\lambda^{k}\right)\\ y^{k+1}=\arg\min L_{\beta }\left(x^{k+1},y;\lambda^{k}\right)(14.2)\\ \lambda^{k+1}=\lambda^{k}-\beta \left(Ax^{k+1}+By^{k+1}-b\right)(14.3) \end{array}\right.$$

The sub-problem (14.1) may be further written as

(15) $$x^{k+1}=\text{argmin}\left\{\theta_{1}\left(x\right)+\frac{\beta }{2}{\left\|Ax+By^{k}-b-\frac{\lambda^{k}}{\beta }\right\|}_{2}^{2}\right\}$$

The sub-problem (14.2) may be further written as

(16) $$y^{k+1}=\text{argmin}\left\{\theta_{2}\left(y\right)+\frac{\beta }{2}{\left\|Ax^{k+1}+By-b-\frac{\lambda^{k}}{\beta }\right\|}_{2}^{2}\right\}$$

Clearly, the ADMM algorithm may divided the original problems into three simple sub-problems. By this splitting technique, the two convex functions are split and the whole solving problem is potentially simplified.

### 2.2.2 Linearized ADMM (L-ADMM) algorithm framework

For the sub-problems (15) and (16) are similar, we will just discuss the linearization technique for (15).

Often, (15) is still hard to solve for usually there is not a simple, close-form solution, for example in case that *θ*_1_ (*x*) is also a quadratic function. Thus, people proposed the LADMM algorithm, whose difference from the ADMM algorithm is only linearization to the quadratic function in (15).

According to the linearization method shown in Section 2.1.4, the linearized form of (15) is

(17) $$x^{k+1}=\text{argmin}\left\{\begin{array}{c} \theta_{1}\left(x\right)+<x,\beta A^{T}\left(Ax^{k}+By^{k}-b-\frac{\lambda^{k}}{\beta }\right)>\\ +\frac{s}{2}{\left\|x-x^{k}\right\|}_{2}^{2} \end{array}\right\}$$

Where, $\text{s}\geq \beta {\left\|A^{T}A\right\|}_{2}=\beta \lambda_{max}\left(A^{T}A\right)$ to guarantee convergence.

(17) is potentially be easier to get a close-form solution.

### 2.2.3 Fully Linearized ADMM (FL-ADMM) algorithm framework

If *θ*_1_ (*x*) is a simple quadratic function, i.e. the corresponding matrix is an identity matrix or is of special structure, for example it is equivalent to a convolution operation, then (17) may get a close-form solution by use of the simple optimality condition and the FFT technique.

However, the matrix corresponding to *θ*_1_ (*x*) usually is not of special structure in CT and EPRI, so (17) is still difficult to get a close-form solution.

The difference of FL-ADMM from L-ADMM is only that it linearizes any quadratic functions. By use of this fully linearization technique, (17) may get a close-form solution.

If $\theta_{1}\left(x\right)=\frac{\gamma }{2}{\left\|Fx-a\right\|}_{2}^{2}$, according to (17) and (10), then (17) has the close-form solution below.


(18) $$x^{k+1}=x^{k}-\frac{\gamma }{s_{1}+s_{2}}\left[F^{T}\left(Fx^{k}-a\right)\right]-\frac{\beta }{s_{1}+s_{2}}\left[A^{T}\left(Ax^{k}+By^{k}-b-\frac{\lambda^{k}}{\beta }\right)\right]$$

Where, ${\text{s}}_{1}\geq \gamma {\left\|F^{T}F\right\|}_{2}$, and ${\text{s}}_{2}\geq \beta {\left\|A^{T}A\right\|}_{2}$.

Clearly, FL-ADMM algorithm may make close-form solution and simplify the difficult sub-problem. Otherwise, one must use time-consuming line search to select the step-size for solving (17) via gradient descent or one must use FFT algorithm to solve (17), for which, *F* and *A* must be of special structure. This proposed FL-ADMM algorithm is the core contribution of this work.

## 2.3 FL-ADMM algorithm instances derivation of three TV models

In this section, we will derive three FL-ADMM algorithm instances corresponding to three TV models.

TV models in image reconstruction have been heavily investigated and have achieved accurate reconstructions via sparse-view projections and/or noisy projections. There are three types of TV models: ucTV, DDcTV and TVcDM models. The unconstrained version is often solved by ADMM algorithm, whereas, DDcTV and TVcDM are often solved by ASD-POCS and CP algorithm. Though the derivation below, we will see that FL-ADMM algorithm may solve any type of TV model and each sub-problem is simple to compute.

Without loss of generality, we derive the algorithm instances for 2D CT.

### 2.3.1 FL-ADMM algorithm for ucTV model

#### (1) The ucTV model

The discrete-to-discrete (D2D) imaging system model of 2D CT may be formulated as

(19) g=Au,

where, *u* is a vector of size *N*, indicating the image, *g* is a vector of size *M*, indicating the projection data, and *A* is the system matrix (projection matrix) of size *M* × *N*, indicating the 2D Radon transform for parallel beam CT and the ray transform for fan beam CT. *A*_*i*,*j*_ is the contribution of the *j*th pixel to the *i*th ray.

If the 2D image is of size [*n*_*x*_, *n*_*y*_], then*N* = *n*_*x*_ × *n*_*y*_. If the projection data (sinogram) is of size [*n*_*p*_, *n*_*a*_], i.e. there are *n*_*a*_ projections and each projection has *n*_*p*_ measurements, then *M* = *n*_*P*_ × *n*_*a*_.

The ucTV model may be formulated as

(20) $$u^{*}=\arg\underset{u}{\min}\frac{1}{2}{\left\|Au-g\right\|}_{2}^{2}+\alpha {\left\|u\right\|}_{\text{TV}},$$

where, ${\left\|u\right\|}_{\text{TV}}$ is the TV norm of image *u*, and its isotropic form is

(21) $${\left\|u\right\|}_{\text{TV}}={\left\|\left({\left\|Du\right\|}_{2}\right)\right\|}_{1}.$$

Here, *D* is a matrix of size 2*N* × *N*, indicating the gradient transform, and is of this form

(22) $$D=\left(\begin{array}{c} D_{x}\\ D_{y} \end{array}\right).$$

Here, *D*_*x*_ and *D*_*y*_ are both matrices of size *N* × *N*, indicating the *x* and *y* direction gradient transform, respectively, shown as

(23) $${\left(D_{x}u\right)}_{x,y}=\left\{\begin{array}{c} u_{x,y}-u_{x-1,y}x\in \left[2,n_{x}\right]\\ 0x=1 \end{array}\right.$$

(24) $${\left(D_{y}u\right)}_{x,y}=\left\{\begin{array}{c} u_{x,y}-u_{x,y-1}y\in \left[2,n_{y}\right]\\ 0y=1 \end{array}\right..$$

Here, *n*_*x*_ and *n*_*y*_ are the row and column number of the image, respectively. And, *x* and *y* are the row and column index of the image, respectively.

Thus, the gradient magnitude transform ${\left\|Du\right\|}_{2}$ may be formulated as

(25) $${\left({\left\|Du\right\|}_{2}\right)}_{x,y}=\sqrt{{{\left(D_{x}u\right)}_{x,y}}^{2}+{{\left(D_{y}u\right)}_{x,y}}^{2}}.$$

And, the TV norm is

(26) $${\left\|u\right\|}_{\text{TV}}=\sum_{x,y}\sqrt{{\left(u_{x,y}-u_{x-1,y}\right)}^{2}+{\left(u_{x,y}-u_{x,y-1}\right)}^{2}}.$$

#### (2) The ucTV-FL-ADMM algorithm

Now, we have the ucTV model,

(27) $$u^{*}=\text{arg}\underset{u}{\text{min}}\frac{1}{2}{\left\|Au-g\right\|}_{2}^{2}+\alpha {\left\|\left({\left\|Du\right\|}_{2}\right)\right\|}_{1}.$$

The FL-ADMM algorithm instance derivation process is as follows.

Let *y* = *Du*, i.e. *Du* − *y* = 0, then (27) is equivalent to this minimization problem,

(28) $$\min\frac{1}{2}{\left\|Au-g\right\|}_{2}^{2}+\alpha {\left\|\left({\left\|y\right\|}_{2}\right)\right\|}_{1}s.t.Du-y=0$$

The corresponding augmented Lagrange function is

(29) $$L_{\beta }\left(u,y;\lambda \right)=\frac{1}{2}{\left\|Au-g\right\|}_{2}^{2}+\alpha {\left\|\left({\left\|y\right\|}_{2}\right)\right\|}_{1}-\langle \lambda ,Du-y\rangle +\frac{\beta }{2}{\left\|Du-y\right\|}_{2}^{2}$$

According to the FL-ADMM algorithm framework, we may derive the algorithm instance.


(30) $$u^{k+1}=\text{arg}\underset{u}{\text{min}}\frac{1}{2}{\left\|Au-g\right\|}_{2}^{2}+\frac{\beta }{2}{\left\|Du-y^{k}-\frac{1}{\beta }\lambda^{k}\right\|}_{2}^{2}$$

We perform linearization to each quadratic function in (30), and get

(31) $$u^{k+1}=\text{arg}\underset{u}{\text{min}}\langle u,A^{T}\left(Au^{k}-g\right)\rangle +\frac{s_{1}}{2}{\left\|u-u^{k}\right\|}_{2}^{2}+\langle u,\beta D^{T}\left(Du^{k}-y^{k}-\frac{1}{\beta }\lambda^{k}\right)\rangle \;+\frac{s_{2}}{2}{\left\|u-u^{k}\right\|}_{2}^{2}$$

Let the gradient of the objective function in (31) be 0, we get

(32) $$u^{k+1}=\frac{\left(s_{1}+s_{2}\right)u^{k}-A^{T}\left(Au^{k}-g\right)-\beta D^{T}\left(Du^{k}-y^{k}-\frac{1}{\beta }\lambda^{k}\right)}{s_{1}+s_{2}}$$

Here, $s_{1}\geq \lambda_{max}\left(A^{T}A\right)$ and $s_{2}\geq \beta \lambda_{max}\left(D^{T}D\right)$. Clearly, (32) is of close form and only involve simple matrix-vector multiplication.


(33) $$y^{\text{k}+1}=\text{arg}\underset{y}{\min}\alpha {\left\|\left({\left\|y\right\|}_{2}\right)\right\|}_{1}+\frac{\beta }{2}{\left\|Du^{k+1}-y-\frac{1}{\beta }\lambda^{k}\right\|}_{2}^{2}$$

According to the 2D shrinkage algorithm in (4), we get

(34) $${\left(y^{\text{k+1}}\right)}_{i,j}={\text{S}}^{2}\left({\left(Du^{k+1}-\frac{1}{\beta }\lambda^{k}\right)}_{i,j},\alpha /\beta \right)$$

Note that *y* and *λ* are vectors of size 2*N*. But, if we regard them as vector-valued vector, then they are both vectors of size *N*. Further, if we consider their 2D form, they are both vector-valued matrices of size [*n*_*x*_, *n*_*y*_]. So, each element of *y* and *λ* is *y*_*i*,*j*_ and *λ*_*i*,*j*_, respectively. But, we should note that they are both a 2D vector, so (34) is a 2D shrinkage algorithm.


(35) $$\lambda^{k+1}=\lambda^{k}-\beta \left(Du^{k+1}-y^{k+1}\right)$$

Now, we get the ucTV-FL-ADMM algorithm.

Algorithm 2

**A.** Pseudocode for *K* steps of the ucTV-FL-ADMM algorithm.


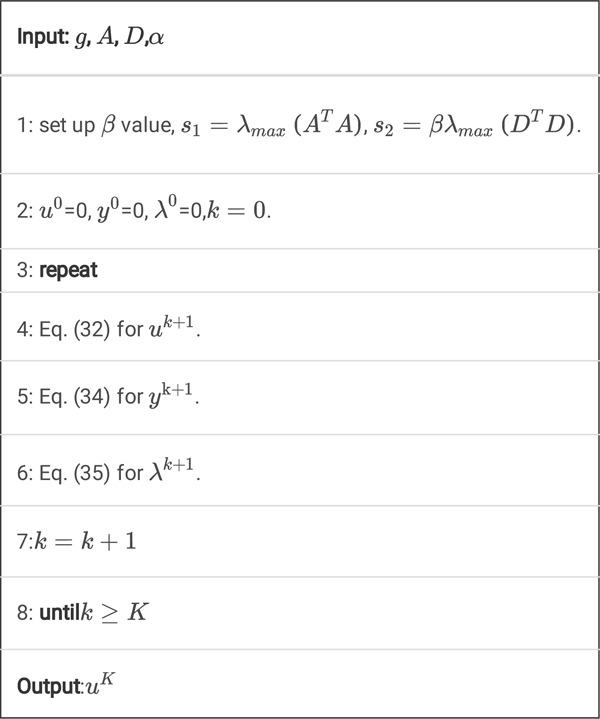


Algorithm 2

A is a simple but effective algorithm. It does not need step-size search via one dimensional search technique. Also, it does not need to use FFT algorithm. The main operations in this algorithm are just simple matrix-vector multiplication and shrinkage algorithm. When we implement this algorithm, the matrices do not need to be explicit form, i.e. we must not store them in the computer memory. We may regard them as some specific operations, for example, means projection operation, whereas means backprojection operation.

However, we find that this algorithm converges very slowly, so we propose an improved algorithm with inner iterations as follows.

Algorithm 2

**B.** Pseudocode for *K* steps of the ucTV-FL-ADMM algorithm with inner iterations.


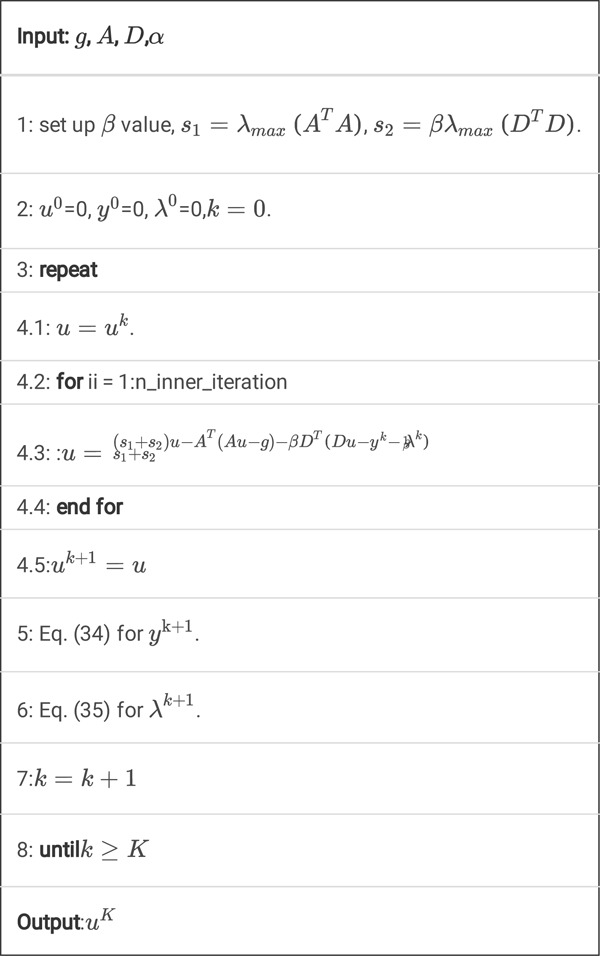


Here, ‘n_inner_iteration’ means the inner iteration number. By adding this inner iterations technique, the algorithm will converge much faster. The reason will be discussed in the Section 4.

### 2.3.2 FL-ADMM algorithm for DDcTV model

#### (1) The DDcTV model

The data divergence constrained, TV (DDcTV) minimization model may be formulated as

(36) $$u^{*}=\text{arg}\underset{u}{\text{min}}{\left\|u\right\|}_{\text{TV}}s.t.{\left\|Au-g\right\|}_{2}\leq \epsilon$$

This model has superior performance than the ucTV model since its model parameter *ϵ* has clear physical meaning that it embodies the noise level and level of system-inconsistence.

#### (2) The DDcTV-FL-ADMM algorithm

According to (21) and (8), (36) may be written as

(37) $$u^{*}=\arg\underset{u}{\min}{\left\|\left({\left\|Du\right\|}_{2}\right)\right\|}_{1}+\delta_{L2Ball\left(\epsilon \right)}\left(Au-g\right)$$

Let *y* = *Du*, and *Au* − *g* = *z*, then (37) is equivalent to this minimization problem,

(38) $$\min{\left\|\left({\left\|y\right\|}_{2}\right)\right\|}_{1}+\delta_{L2Ball\left(\epsilon \right)}\left(z\right)\;\text{s}.\text{t}.\;Du-y=0\text{and}Au-z=g$$

The corresponding augmented Lagrange function is

(39) $$L_{\beta_{1},\beta_{2}}\left(u,y,z;\lambda_{1},\lambda_{2}\right)={\left\|\left({\left\|y\right\|}_{2}\right)\right\|}_{1}+\delta_{L2Ball\left(\epsilon \right)}\left(z\right)-\langle \lambda_{1},Du-y\rangle +\frac{\beta_{1}}{2}{\left\|Du-y\right\|}_{2}^{2}\;-\langle \lambda_{2},Au-z-g\rangle +\frac{\beta_{2}}{2}{\left\|Au-z-g\right\|}_{2}^{2}$$

According to the FL-ADMM algorithm framework, we may derive the algorithm instance.


(40) $$u^{k+1}=\text{arg}\underset{u}{\text{min}}\frac{\beta_{1}}{2}{\left\|Du-y^{k}-\frac{1}{\beta_{1}}\lambda_{1}^{k}\right\|}_{2}^{2}+\frac{\beta_{2}}{2}{\left\|Au-z^{k}-g-\frac{1}{\beta_{2}}\lambda_{2}^{k}\right\|}_{2}^{2}$$

By linearizing the two quadratic functions in (40), letting the gradient of the objective function be 0, we may get

(41) $$u^{k+1}=u^{k}-\frac{\beta_{1}}{s_{1}+s_{2}}D^{T}\left(Du^{k}-y^{k}-\frac{1}{\beta_{1}}\lambda_{1}^{k}\right)-\frac{\beta_{2}}{s_{1}+s_{2}}A^{T}\left(Au^{k}-z^{k}-g-\frac{1}{\beta_{2}}\lambda_{2}^{k}\right)$$

Here, $s_{1}\geq \beta_{1}\lambda_{max}\left(D^{T}D\right)$ and $s_{2}\geq \beta_{2}\lambda_{max}\left(A^{T}A\right)$. Cleary, (41) is of a simple closed-form and thus is easy to implement.


(42) $$y^{\text{k}+1}=\text{arg}\underset{y}{\min}{\left\|\left({\left\|y\right\|}_{2}\right)\right\|}_{1}+\frac{\beta_{1}}{2}{\left\|Du^{k+1}-y-\frac{1}{\beta_{1}}\lambda_{1}^{k}\right\|}_{2}^{2}$$

According to the 2D shrinkage algorithm in (4), we get

(43) $${\left(y^{\text{k}+1}\right)}_{i,j}={\text{S}}^{2}\left({\left(Du^{k+1}-\frac{1}{\beta_{1}}\lambda_{1}^{k}\right)}_{i,j},\frac{1}{\beta_{1}}\right)$$

(44) $$z^{\text{k}+1}=\text{arg}\underset{z}{\min}\delta_{L2Ball\left(\epsilon \right)}\left(z\right)+\frac{\beta_{2}}{2}{\left\|Au^{k+1}-z-g-\frac{1}{\beta_{2}}\lambda_{2}^{k}\right\|}_{2}^{2}\;=\text{P}\left(Au^{k+1}-g-\frac{1}{\beta_{2}}\lambda_{2}^{k},L2Ball\left(\epsilon \right)\right)=\frac{\epsilon \left(Au^{k+1}-g-\frac{1}{\beta_{2}}\lambda_{2}^{k}\right)}{\max\left(\epsilon ,{\left\|Au^{k+1}-g-\frac{1}{\beta_{2}}\lambda_{2}^{k}\right\|}_{2}\right)}$$

(45) $$\lambda_{1}^{k+1}=\lambda_{1}^{k}-\beta_{1}\left(Du^{k+1}-y^{k+1}\right)$$

(46) $$\lambda_{2}^{k+1}=\lambda_{2}^{k}-\beta_{2}\left(Au^{k+1}-z^{k+1}-g\right)$$

Now, we get the DDcTV-FL-ADMM algorithm.

Algorithm 3

**A.** Pseudocode for *K* steps of the DDcTV-FL-ADMM algorithm.


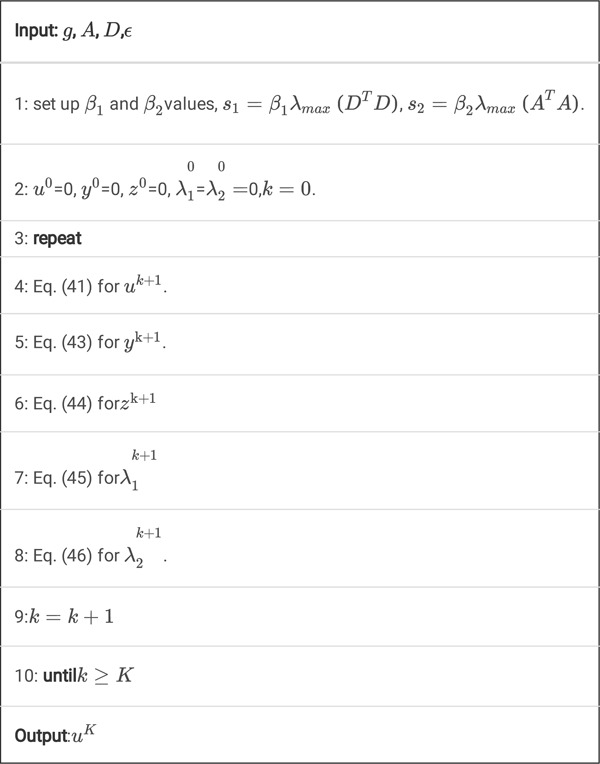


Also, this standard FL-ADMM algorithm is slow to convergence, so we propose the fast, improved FL-ADMM algorithm with inner iterations.

Algorithm 3

**B.** Pseudocode for *K* steps of the DDcTV-FL-ADMM algorithm with inner iterations.


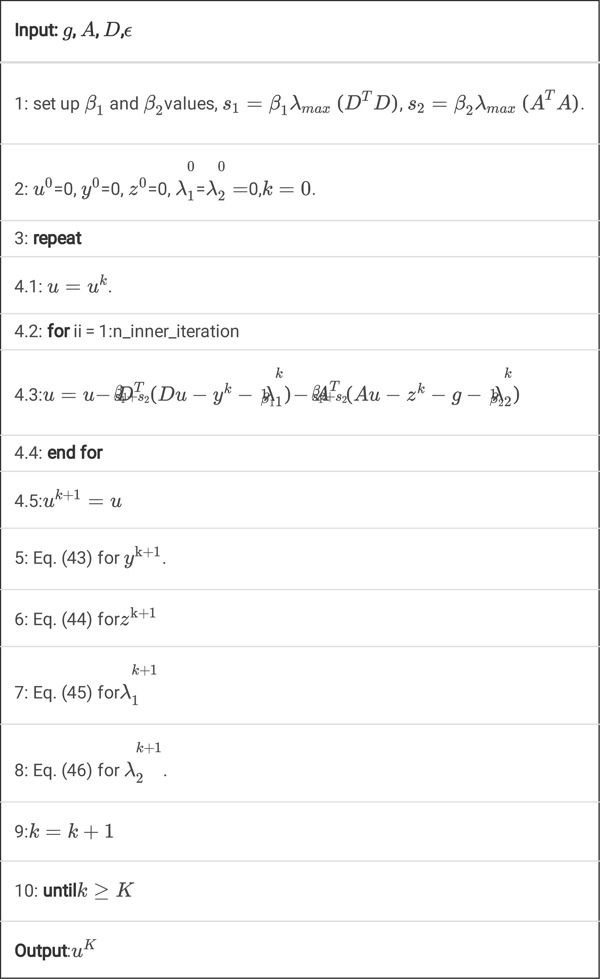


By adding this inner iterations technique, the algorithm will converge much faster. The reason will be discussed in the Section 4.

### 2.3.3 FL-ADMM algorithm for TVcDM model

#### (1) The TVcDM model

The TV constrained, data divergence minimization (TVcDM) algorithm is another constrained TV model and has been widely used in CT and EPRI. They are often solved by the CP algorithm. Next, we will see that it may also be solved by FL-ADMM and will see that its derivation is easier than that of CP algorithm for it does not need to calculate the convex conjugate functions.

The TVcDM model may be formulated as

(47) $$u^{*}=\arg\underset{u}{\min}\frac{1}{2}{\left\|Au-g\right\|}_{2}^{2}s.t.{\left\|u\right\|}_{\text{TV}}\leq t$$

#### (2) The TVcDM-FL-ADMM algorithm

By use of the indicator function and the definition of the TV norm, (47) is equivalent to

(48) $$u^{*}=\arg\underset{u}{\min}\frac{1}{2}{\left\|Au-g\right\|}_{2}^{2}+\delta_{L1Ball\left(t\right)}\left({\left\|Du\right\|}_{2}\right)$$

Let *y* = *Du*, then (48) becomes

(49) $$\min\frac{1}{2}{\left\|Au-g\right\|}_{2}^{2}+\delta_{L1Ball\left(t\right)}\left({\left\|y\right\|}_{2}\right)\;s.t.Du-y=0$$

Then, the corresponding augmented Lagrange function is

(50) $$L_{\beta }\left(u,y;\lambda \right)=\frac{1}{2}{\left\|Au-g\right\|}_{2}^{2}+\delta_{L1Ball\left(t\right)}\left({\left\|y\right\|}_{2}\right)-\langle \lambda ,Du-y\rangle +\frac{\beta }{2}{\left\|Du-y\right\|}_{2}^{2}$$

According to the FL-ADMM algorithm framework, we may derive the corresponding algorithm instance.


(51) $$u^{k+1}=\arg\underset{u}{\min}\frac{1}{2}{\left\|Au-g\right\|}_{2}^{2}+\frac{\beta }{2}{\left\|Du-y^{k}-\frac{1}{\beta }\lambda^{k}\right\|}_{2}^{2}$$

It is the same as (30). By full linearization, we may get its close solution.


(52) $$u^{k+1}=\frac{\left(s_{1}+s_{2}\right)u^{k}-A^{T}\left(Au^{k}-g\right)-\beta D^{T}\left(Du^{k}-y^{k}-\frac{1}{\beta }\lambda^{k}\right)}{s_{1}+s_{2}}$$

Here, $s_{1}\geq \lambda_{max}\left(A^{T}A\right)$ and $s_{2}\geq \beta \lambda_{max}\left(D^{T}D\right)$.


(53) $$y^{k+1}=\arg\underset{y}{\min}\delta_{L1Ball\left(t\right)}\left({\left\|y\right\|}_{2}\right)+\frac{\beta }{2}{\left\|Du^{k+1}-y-\frac{1}{\beta }\lambda^{k}\right\|}_{2}^{2}$$

(54) $$m=\text{P}\left({\left\|Du^{k+1}-\frac{1}{\beta }\lambda^{k}\right\|}_{2},L1Ball\left(t\right)\right)$$

(55) $${\left(y^{k+1}\right)}_{i,j}=\frac{{\left(Du^{k+1}-\frac{1}{\beta }\lambda^{k}\right)}_{i,j}}{{\left({\left\|Du^{k+1}-\frac{1}{\beta }\lambda^{k}\right\|}_{2}\right)}_{i,j}}\times m_{i,j}$$

(56) $$\lambda^{k+1}=\lambda^{k}-\beta \left(Du^{k+1}-y^{k+1}\right)$$

Now, we get the TVcDM-FL-ADMM algorithm.

Algorithm 4

A. Pseudocode for K steps of the TVcDM-FL-ADMM algorithm.


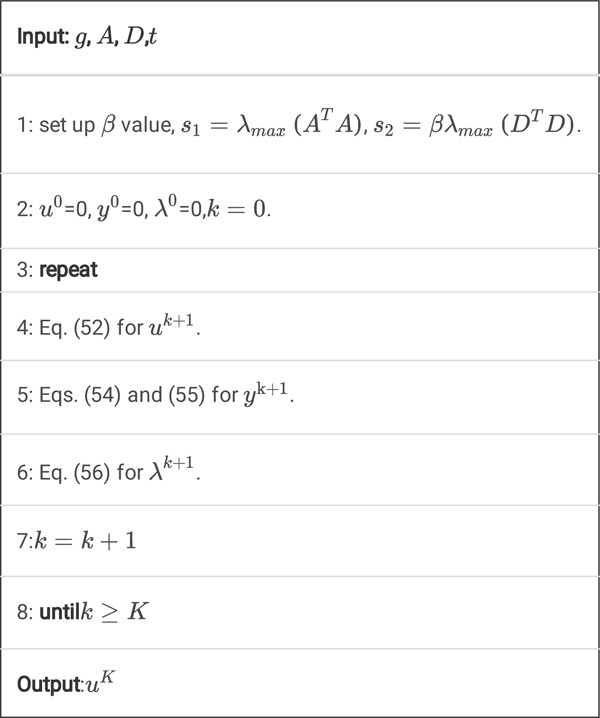


Also, we may speed up this algorithm by use of inner iteration technique.

Algorithm 4

**B.** Pseudocode for *K* steps of the TVcDM-FL-ADMM algorithm with inner iteration.


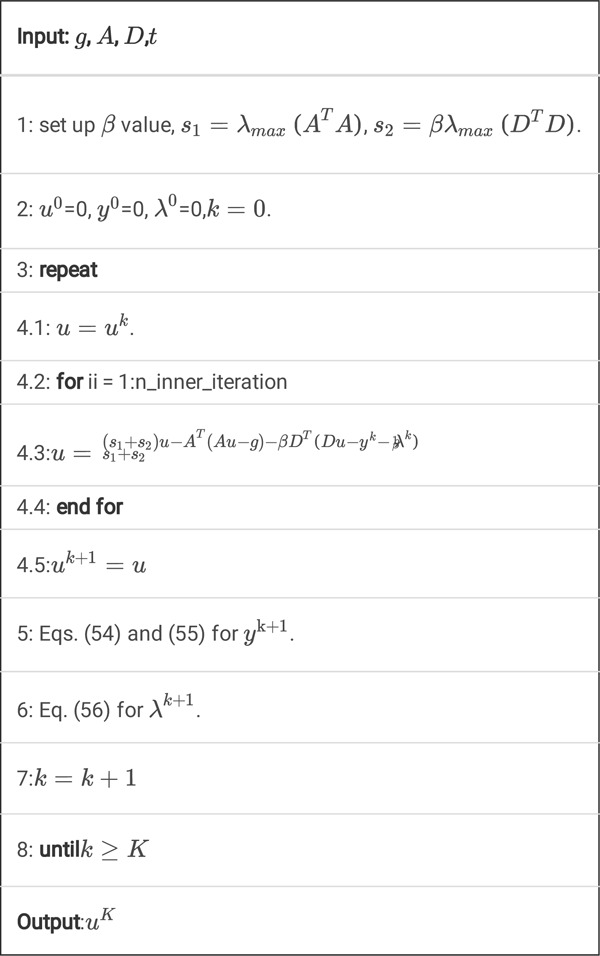


By adding this inner iterations technique, the algorithm will converge much faster. The reason will be discussed in Section 4.

## 3. Results

The aim of this work is to design a FL-ADMM algorithm framework and to give prototyping method for optimization models in image reconstruction via the FL-ADMM algorithm framework.

For optimization based image reconstruction, we know that optimization model decides the final solution, whereas the solving algorithm just impact the convergence rate and path. Since the FL-ADMM algorithm is a solving algorithm, we should validate if it may solve optimization model accurately, and evaluate what may impact its convergence rate.

We have derived three FL-ADMM algorithms for ucTV, DDcTV and TVcDM models. Next, we will validate and evaluate this solving algorithm for DDcTV model in 2D CT, no loss of generality.

We design 4 studies: (1) Inverse crime of DDcTV-FL-ADMM algorithm; (2) The impact of *β* selection on convergence rate; (3) The impact of inner iteration number on convergence rate; and (4) Comparison with the CP algorithm.

### 3.1. Inverse crime of DDcTV-FL-ADMM algorithm

Inverse crime is a tool to validate the correctness of an inverse problem [41]. If the projection data is complete and exact, then the sign of inverse crime is that the reconstructed image is almost the same with the truth image, i.e. there is not any error except for the computer floating point error.

The imaging configurations are as follows. The phantom is the Shepp-Logan phantom of size [256, 256]. The imaging coordinate system is located at [128, 128]. The projection signal at each angle is of size 256 and its coordinate range is [−127, 128]. The virtual detector bin length is 1. The pixel size is also 1. The parallel beam scanning pattern is adopted. The projections are simulated by use of pixel-driven method [42]. We collect 256 projections uniformly distributed in the range of [0, *π*].

Thus, the projection data is of size [256, 256] and the image is also of size [256, 256]. For this discrete-to-discrete imaging linear system, the number of unknowns and the number of equations are the same. For the linear system is absolutely consistent, the solution of the corresponding DDcTV model should be accurate enough to reappear the phantom, i.e. the RMSE of the reconstructed image compared with the truth image should be small enough.

If the inverse crime may be achieved, then we may think that the imaging system modelling, the optimization problem modelling, the FL-ADMM algorithm derivation and its computer implementation are all correct.

For this reconstruction case, we use Algorithm 3-B (it is used in all reconstructions in Section 3), in which, *ϵ* = 0, *β*_1_ = *β*_2_ = 1, and the inner iteration number is 50. The gray image has only 256 gray-level. So, if RMSE is less than 1/256 ≈ 3.9 × 10^−3^, the reconstructed image and the truth image will be visually the same, which may be the sign of inverse crime. More strictly, we define the sign of inverse crime as that RMSE ≤ 10^−4^.

**Figure 3:**
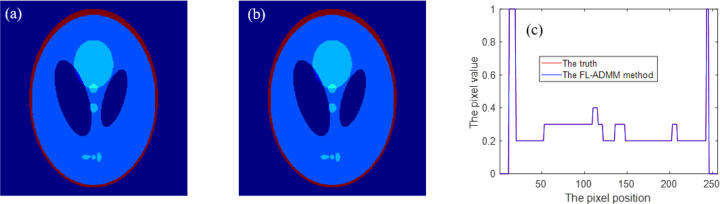
The reconstructed image and profile of the DDcTV-FL-ADMM algorithm in the context of inverse crime. (a) is the truth image, (b) is the reconstructed image, and (c) plots the vertical-center-line profiles of (a) and (b).

**Fig. 4:**
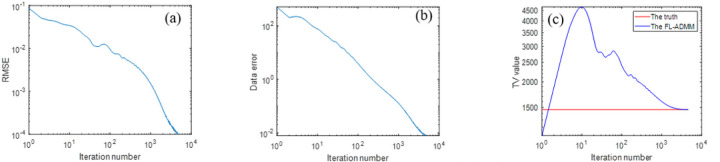
The iteration trends of the DDcTV-FL-ADMM algorithm in the context of inverse crime. (a) is for the RMSE of the reconstructed image. (b) is for the data error, i.e. ${\left\|Au-g\right\|}_{2}$. (c) is for the TV value of the reconstructed image.

At iteration 4570, inverse crime is achieved. The reconstructed images and the corresponding profiles are shown in Fig. 3. Three iteration trends are plotted in Fig. 4.

From Fig. 3, we may see that the reconstructed image is visually the same with the truth image, and that the vertical-center-line profiles of the reconstructed image and the truth image are completely coincident. This shows that inverse crime is achieved. Now, we may think that the imaging system model, the DDcTV model, the FL-ADMM algorithm and its computer implementation are all correct.

In Fig. 4, we plots three iteration trends to observe the iteration behavior. It may be seen that the RMSE of the reconstructed image and the data error between the guessed data and the truth data may go down and down with the increase of iteration number. At iteration 4570, the RMSE has been less than 10^−4^, which means the inverse crime is achieved. We may see that these curves still has a descent trend, showing its good convergence performance. From Fig. 4 (c), we may see that during the later iteration period, the TV value may go down and down with the iteration marching. When the iteration stops, the TV value of the reconstructed image becomes the same with the truth TV.

### 3.2. The impact of *β* selection on convergence rate of the DDcTV-FL-ADMM algorithm

In this section, we evaluate how the penalty parameter, *β*, impact convergence rate. Here,*β* = *β*_1_ = *β*_2_.

The simulation phantom is the FORBILD phantom [43] of size [256, 256]. The projection data is of size [256, 100]. The scanning pattern is of parallel beam form. The 100 projections are uniformly distributed in the range of [0, *π*]. In the DDcTV-FL-ADMM reconstructions, we vary *β* from 0.01, 0.1, 1, 10, to 100, fix the inner iteration number as 50, and fix the iteration number as 5000 to evaluate their respective convergence rate.

**Figure 5:**
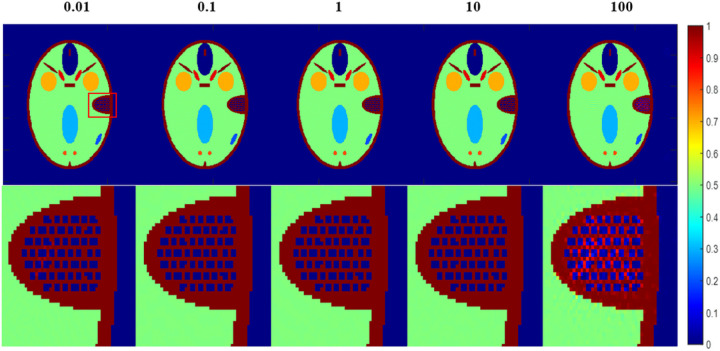
The reconstructed images of the DDcTV-FL-ADMM algorithm with different β. The number above the images indicate the β value. The images on the second row is the zoomed-in images in the red box shown in the first row. The display window is [0,1].

**Figure 6:**
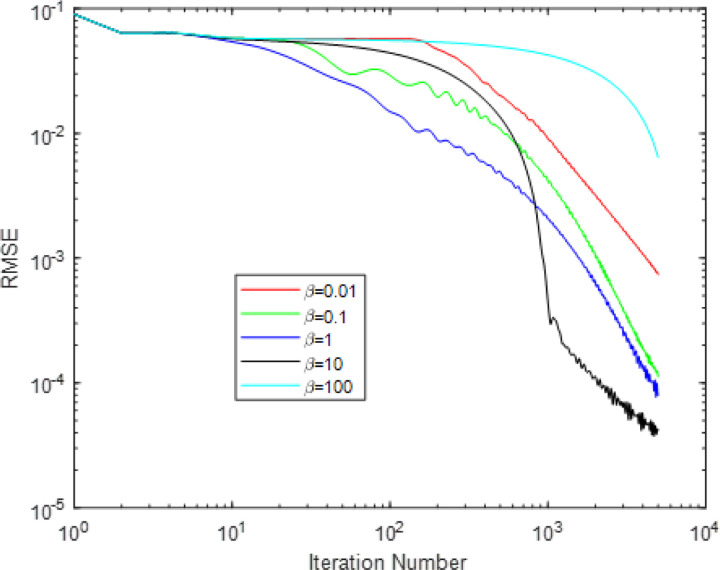
Plots of RMSE as function of iteration number. The iteration number is 5000. The two axis are both of logarithm form.

The reconstructed images of different *β* are shown in Fig. 5, and the convergence curves of different *β* are shown in Fig. 6.

All the reconstructions of different *β* are stopped at iteration 5000. Thus, more accurate images means faster convergence. From Fig. 5, we may see that *β* values of 0.1, 1 and 10 may achieve more accurate reconstructions, whereas *β* values of 0.01 and 100 lead to reconstructions of a certain level of artifacts. From the image at row 2 and column 5, we may see obvious artifacts. This means that the FL-ADMM algorithm of *β* value of 100 suffer from the slowest convergence rate. This observation may be more clearly seen in Fig. 6. The order of convergence rate of different *β* from slow to fast is 100, 0.01, 0.1, 1, and then 10. This indicates that an appropriate *β* value may achieve fast convergence, whereas too large or too small values always lead to too slow convergence. Even that too huge value may lead to a wrong convergence, i.e. the RMSE will converge to a very large value. However, we want to emphasize that the optimal *β* value is imaging-condition dependent. In this simulation study, the optimal value is 10. But, in other cases, it may be other value. Usually, one may vary the *β* value with interval of an order of magnitudes, then select the optimal one to achieve the fastest convergence.

### 3.3. The impact of inner iteration number on convergence rate of the DDcTV-FL-ADMM algorithm

In Algorithm 3-B, i.e. the DDcTV-FL-ADMM algorithm, there is an inner loop process. In this section, we investigate how the inner iteration number impact the convergence rate.

**Figure 7:**
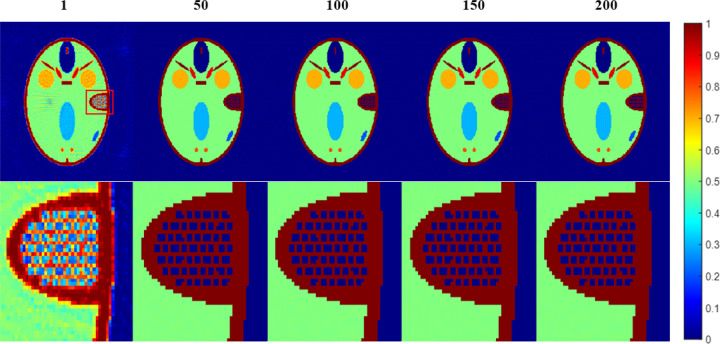
The reconstructed images of the DDcTV-FL-ADMM algorithm with different inner iteration number. The number above the images indicate the inner iteration number. The images on the second row is the zoomed-in images in the red box shown in the first row. The display window is [0,1].

**Figure 8:**
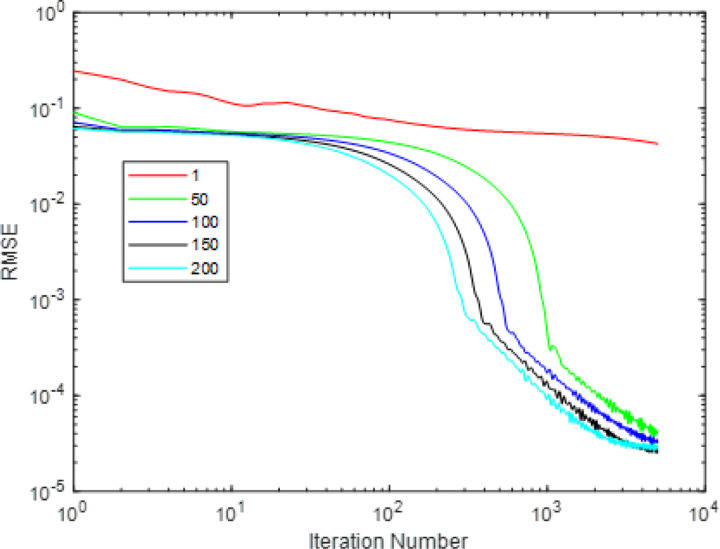
Plots of RMSE as function of iteration number. The iteration number is 5000. The two axis are both of logarithm form.

The phantom is still the FORBILD phantom of size [256, 256]. The imaging condition is the same with that of Section 3.2. In the DDcTV-FL-ADMM reconstructions, we vary the inner iteration number from 1, 50, 100, 150, to 200 and fix the iteration number as 5000, fix *β* as 10, to evaluate their respective convergence rate. Figure 7 shows the reconstructed images and Fig. 8 plots the convergence curves

From Fig. 7, we may see that the reconstructed images with inner iteration number of 50, 100,150 and 200 all have higher accuracy. However, if the inner iteration number is 1, i.e. if the FL-ADMM algorithm used is Algorithm 3-A, the reconstructed image suffers from serious artifacts. This means that appropriately selected inner iteration number may achieve faster convergence. The standard FL-ADMM algorithm without inner iteration always leads to too slow convergence rate. This observation may be clearly seen in Fig. 8. Though use of larger inner iteration number may achieve faster convergence rate, the inner loop process will take longer time. Thus, one should select an appropriate inner iteration number to achieve fast convergence and fast inner loop computation. In this case, inner iteration number of 50 is the optimal selection. Also, we want to emphasize that the optimal number of inner iteration is imaging-condition dependent. Usually, one may select some values to perform reconstructions and then select the optimal one.

### 3.4 Comparison with the CP algorithm

CP algorithm has been proposed and widely used in image reconstruction for more than 10 years. We have applied the CP algorithm in EPR imaging and CT. The most important advantage of the CP algorithm is that it may always get closed-form solutions for sub-problems and the finial algorithm instance only involves simple matrix-vector multiplications and some simple operations. Thus, we say the CP algorithm is a fast prototyping tool for optimization based image reconstruction. Similar to CP, the FL-ADMM is also a fast prototyping tool for it may always get closed-form solutions for difficult sub-problems and it has not special demand on system matrix and sparse-transform matrix.

Through the studies in subsections 3.1 to 3.3, we have known the correctness of the FL-ADMM algorithm and have realized how the *β* selection and inner iteration number impact the convergence rate. In this subsection, we investigate the sparse reconstruction capability of the DDcTV-FL-ADMM algorithm by comparison with the DDcTV-CP algorithm whose pseudocode and some explanations are shown in Appendix 1.

The phantom is a real thoracic CT image of size [256, 256]. The imaging condition is the same with that of Section 3.2. In the DDcTV-FL-ADMM reconstructions, we fix the inner iteration number as 50, fix *β* as 10, and vary the projection number from 20,40,60,80, to 100. In the DDcTV-CP reconstruction, we set *λ* = 1 and $\nu =\frac{{\left\|A\right\|}_{2}}{{\left\|D\right\|}_{2}}$ and vary the projection number from 20, 40, 60, 80, to 100.

**Figure 9:**
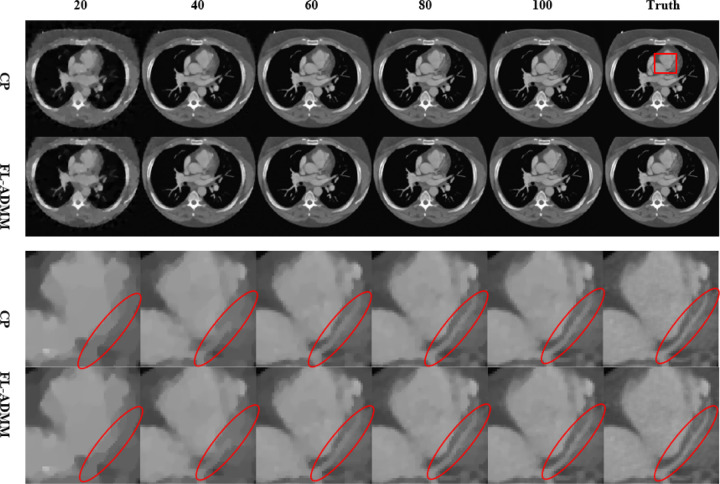
The reconstructed images by the DDcTV-CP and DDcTV-FL-ADMM algorithms. The number above the images indicate the projection number. The text at the left of the images indicate the algorithm used. The images in row 3 and 4 are the enlarged region-of-interest (ROI) images which is indicated by the red rectangle in the right-top image. The red ellipses encircle a fine structure to emphasize observation.

Figure 9 The reconstructed images by the DDcTV-CP and DDcTV-FL-ADMM algorithms. The number above the images indicate the projection number. The text at the left of the images indicate the algorithm used. The images in row 3 and 4 are the enlarged region-of-interest (ROI) images which is indicated by the red rectangle in the right-top image. The red ellipses encircle a fine structure to emphasize observation.

The two algorithms are solving the same optimization model, DDcTV model. Theoretically speaking, model decides the solution, whereas the solving algorithm only decides the convergence rate and path. However, practically speaking, different solving algorithms cannot achieve the absolutely the same solution because the model-solution may be a solution-set, the algorithm-parameters cannot be selected absolutely optimally and there always exists numerical error induced by the computer floating point error. If the two solvers are both convergent accurate solvers, their reconstruction accuracy should be both very high and should be very similar. The CP algorithm has been used in CT and other imaging modalities for more than ten years and has been deeply explored. In this comparison, we may regard the CP algorithm as the state-of-the-art (SOTA) algorithm.

From Fig. 9, we may see that the image quality is better and better with the increase of the projection number for both algorithms. The reconstructed images via 100 projection by the two algorithms are both almost the same with the truth image. With the decrease of the projection number, the reconstructed images degrade gradually. The reconstructed images via 20 projections become too smooth. Their ROI images have lost the fine structure. For the two algorithms, they are both accurate solvers of the DDcTV model and both have capability to perform accurate sparse reconstructions. Comparing the two algorithms by Fig. 9, we almost cannot see the difference between each other. This shows the FL-ADMM may achieve comparable reconstruction quality with the SOTA CP algorithm according to the visual observations.

Table 1 to 3 shows the quantitative comparison results of the two algorithms via metric of RMSE, SSIM and PSNR, respectively. RMSE means root mean square error, SSIM means structural similarity index measure, and PSNR means peak signal to noise ratio. From them, we may see that the two algorithms have very close accuracy, which is visually validated by Fig. 9. However, we may also see that the FL-ADMM algorithm is always a little bit better than the CP algorithm. This is because the optimal *β* selection in FL-ADMM is easier than the optimal selections of *λ* and *ν* in CP algorithm. For *β*, one may just search the optimal value via several reconstructions of different values. However, for the optimal selections of *λ* and *ν*, it would be more difficult for the search is in the range of a two dimensional plane. In image reconstructions, we usually search these values at tenfold intervals. Clearly, one-algorithm-parameter selection is much easier than two-algorithm-parameters selections. Viewed from this perspective, FL-ADMM algorithm is superior to the CP algorithm.

Both qualitative and quantitative evaluations show that the FL-ADMM algorithm may accurately solve the DDcTV model. In fact, we have also evaluated the FL-ADMM algorithms for ucTV and TVcDM models. To be brief, we just show the results of DDcTV-FL-ADMM algorithm. All the experiments on Shepp-Logan, FORBILD and real-CT-image phantoms show that FL-ADMM algorithm is a simple, effective, convergent and universal solving algorithm for optimization models in image reconstruction.

## 4. Discussions

In this work, we propose a novel ADMM algoithm, FL-ADMM algorithm, which may be used as a prototyping tool for optimization model in image reconstruction. The key operation is to expand all the quadratic terms so that the corresponding sub-problem may get a simple closed-form solution. Further, we propose the fast FL-ADMM algorithm by use of the inner iteration technique. We have derived three FL-ADMM algorithm instances for three TV models, ucTV, DDcTV, and TVcDM. Further, we validate and evaluate the correctness and sparse recontruction capability of the DDcTV-FL-ADMM algorithm. Also, we analyze how the penalty parameter and the inner iteration number impact the convergence rate. In addition, we compare this algorithm with the SOTA CP algorithm and discuss its potential superioty.

In optimization based image reconstruction, especially in TV-type norm based image reconstruciton, the ADMM algorithm always has a problem that one sub-problem has not simple closed-form solution. Usually, people has two choices to solve this problem. One is to use gradient desent algorithm to solve this sub-problem. However, the step-size selection is difficult. If one uses the line search method to select the optimal step-size for each iteration, it needs too long time, especially when the imaging model is large scale. The other one is to use FFT technique. In fact, why FFT may be used is because the sparse transform matrix and the system matrix may both be regarded as a convolution operation. So, this method has not universality. Once a sparse transform cannot be regarded as a convolution operation, this method loses efficacy.

These difficuties of ADMM motivated this work whose aim is to devise a method to simplify the implementation of the ADMM algoirthm. Motivated by the linearization technique of the L-ADMM, we propose the FL-ADMM algorithm and propose its acceleration version.

In Section 2, the standard FL-ADMM algorithms are named Algorithm-A, whearas the acclerated FL-ADMM algorithm are named Algoritm-B. For example, for the DDcTV-FL-ADMM algorithm, the standard algorithm is Algorithm 3-A, whereas its improved, accelerated version is Algorithm 3-B.

In fact, the accelerated FL-ADMM algorithm uses a special gradient desenct algorithm. In Algorithm 3-B, Eq. (4.3) is, in fact, a gradient desent equation. But, very importantly, the step-size is $\frac{1}{s_{1}+s_{2}}$, which may be calculated before the whole iteration process. Thus, compared with the ordinary gardient descent algorihtm, this proposed method will be much faster for it doesnot need the time-consuming and complicated step-size search via line search technique.

Compared to the ADMM algorithm using FFT, this proposed algoirthm has universality for it does not need that the sparse transform matrix and the system maxtrix may be both regarded as a convolution operation.

FL-ADMM algorithm is a uinversal optimization algorithm which may be used for solving optimiztion models, especially TV-type models in image reconstruction. It is simple, effective, convergent and universal. ‘Simple’ means the main operations in the final algorithm instance only involve simple matrix-vector multiplications and simple shrinkage or projection operations. ‘Effective’ means it is actually the solver of the optimization model. ‘Convergent’ means it may achieve the solutions of the optimizaiton model. ‘Universal’ means it must not demand that the system matrix and/or the sparse transform matrix have special structures.

Still, we do not know why the mathematically-derived, standard FL-ADMM without inner iteration is so slow. When we observed that the closed-form solution for the sub-problem on data-fidelity term, for examle, Eq. (52) in Algorithm 3-A, is, in fact, a gradient descent step, we realize that the slow convergence may be because Eq. (52) only runs one time of gradient descent. Thus, we propose to accelerate the standard FL-ADMM algorithm by letting the gradient descent steps run more times/iterations. Though, we use inner iteration to perform gradient descent here, it is different from the direct use of gradient descent algorithm to solve the corresponding sub-problem, which needs time-consuming line search to select the optimal step-size for each iteration.

In the Results Section, we only compare the FL-ADMM algorithm with the SOTA CP algorithm and do not compare it with other ADMM-type algorithm. This is because the aim of this work is to design solving algorithm which should be simple and universal. The ADMM + line-search algorithm is not simple for it needs line search for each iteration in difficult-sub-problem iterations. The ADMM + FFT algorithm is not universal for it demands the special structure of the sparse transform. Compared with these two types of algorithms, viewed from the perspective of simplicity and universality, the superiority of the FL-ADMM algorithm is clear. The CP algorithm should be compared for it is also a solver that is simple and universal. It may solve convex optimization model, no matter it is smooth or non-smooth. Similar to FL-ADMM, the CP algorithm only involve simple matrix-vector multiplications and shrinkage/projection operations and has not special demands on the system matrix and sparse transform matrix. The CP algorithm has been explored in image reconstructions for many years, so we regard it as SOTA. The comparisons show the FL-ADMM algorithm may achieve higher accuracy because of the easier algorithm-parameters selections.

Though the FL-ADMM algorithm and CP algorithm have similar advantages, i.e. the simplicity and universality, the newly proposed FL-ADMM algorithm may have more advantages. For CP algorithm, the calculation of the convex conjugate functions are necessary, whereas the FL-ADMM algorithm only involves the original terms in the optimization model. For some models, the corresponding convex conjugate functions may be difficult to solve. However, the FL-ADMM algorithm instance may be very easy. For example, for the L(1/2) norm based TpV minimization model, the convex conjugate function of the L(1/2) norm may be difficult to solve. But, in FL-ADMM algorithm instance, the sub-problem involving the L(1/2) norm may be easily solved by *half* shrinkage operation [44]. Thus, the FL-ADMM algorithm, on the one hand, improved the classical ADMM and L-ADMM algorithm, on the other hand, may solve some special optimization model which may be difficult to solve via CP algorithm.

Just like the CP algorithm, the FL-ADMM algorithm is not so fast. So, in the future, research on how to speed up this algorithm is necessary. However, this is out of the scope of this work. Just like CP algorithm is a fast prototyping tool for optimization model because of its simplicity and universality, the FL-ADMM algorithm is also a fast prototyping tool. Once one designs a new optimization model for image reconstruction, he/she may derive the FL-ADMM algorithm instance quickly and begin to evaluate the performance of this new model. This is the meaning of fast prototyping tool in optimization based image reconstruction.

## 5. Conclusions

The conclusions of this work may be summed up briefly as the following points.

1. The FL-ADMM algorithm is a universal, simple, effective, and accurate solver of convex optimiztion model in image reconstruction, no matter it is unconstrained or constrained.
2. The FL-ADMM algoirthm improves the traditional ADMM algorithm by avoiding the step-size search for gradient desecnt and the special demands on the sparse transfrom and the system matrix for use of FFT technique.
3. The penalty parameter in this proposed algoritm may impact the convergence rate. Too large or small values both lead to slow convergence.
4. The inner iteration number in this algorithm may impact the convergence rate. One may select the optimal one by running several reconstrutions with different inner iteration number. According to our experience, 30–50 is efficient. But, we note that the optimal inner iteration number is imaging-condition dependent.
5. Compared to the CP algorithm, its algorithm-parameters are easy to tune and thus may achieve higher accuracy.
6. Compared to the CP algorithm, it may solve some special optimization model which is difficult for CP because of the use of convex conjugate functions.

In the future, the FL-ADMM algorithm should be focused on its acceleration technique which may borrow the ideas in accelertion of the traditional or linearized ADMM algorithms.

## Supplementary Material

Supplement 1

## Figures and Tables

**Fig. 1:**
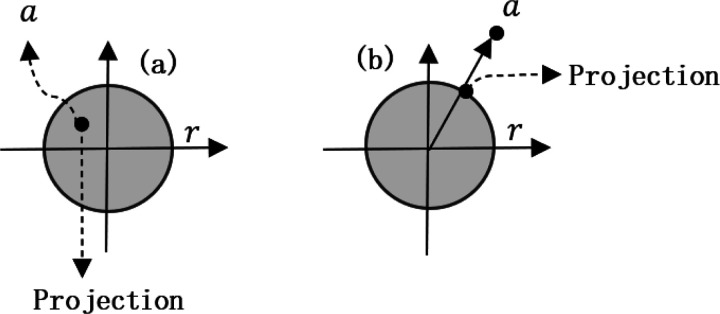
Projection onto *ℓ*_2_ norm ball. (a) is for the case that *a* is in the ball, and (b) is for the case that *a* is outside the ball.

**Table 1:** RMSE comparison of the FL-ADMM and CP algorithms

	20	40	60	80	100
CP	0.0451	0.0225	0.0158	0.0127	0.0107
FL-ADMM	**0.0445**	**0.0214**	**0.0145**	**0.0108**	**0.0082**

**Table 2:** SSIM comparison of the FL-ADMM and CP algorithms

	20	40	60	80	100
CP	0.800	0.928	0.962	0.975	0.981
FL-ADMM	**0.806**	**0.934**	**0.966**	**0.980**	**0.987**

**Table 3:** PSNR comparison of the FL-ADMM and CP algorithms

	20	40	60	80	100
CP	26.92	32.96	36.02	37.95	39.38
FL-ADMM	**27.02**	**33.40**	**36.80**	**39.34**	**41.67**
