# Supplementary material for: A Simple but Universal Fully Linearized ADMM Algorithm for Optimization Based Image Reconstruction
Source: Res Sq. 2023 Apr 28:rs.3.rs-2857384. Preprint. [Version 1] doi: 10.21203/rs.3.rs-2857384/v1 (PMC10168464; doi:10.21203/rs.3.rs-2857384/v1)
Supplement: Supplement 1 [file NIHPPrs2857384v1-supplement-1.pdf]

Supplementary Files

This is a list of supplementary files associated with this preprint. Click to download.

- Appendix1.docx
